# Novel Biomimetic Human TLR2-Derived Peptides for Potential Targeting of Lipoteichoic Acid: An In Silico Assessment

**DOI:** 10.3390/biomedicines9081063

**Published:** 2021-08-21

**Authors:** Nikita Devnarain, Ayman Y. Waddad, Beatriz G. de la Torre, Fernando Albericio, Thirumala Govender

**Affiliations:** 1 Discipline of Pharmaceutical Sciences, College of Health Sciences, University of KwaZulu-Natal, Durban 4000, South Africa; DevnarainN@ukzn.ac.za; 2 Peptide Science Laboratory, School of Chemistry and Physics, University of KwaZulu-Natal, Durban 4001, South Africa; Albericio@ukzn.ac.za; 3 KRISP, School of Laboratory Medicine and Medical Sciences, College of Health Sciences, University of KwaZulu-Natal, Durban 4001, South Africa; Garciadelatorreb@ukzn.ac.za

**Keywords:** TLR2-derived peptides, biomimetic, human Toll-like receptor 2, lipoteichoic acid

## Abstract

Antimicrobial resistance is one of the most significant threats to health and economy around the globe and has been compounded by the emergence of COVID-19, raising important consequences for antimicrobial resistance development. Contrary to conventional targeting approaches, the use of biomimetic application via nanoparticles for enhanced cellular targeting, cell penetration and localized antibiotic delivery has been highlighted as a superior approach to identify novel targeting ligands for combatting antimicrobial resistance. Gram-positive bacterial cell walls contain lipoteichoic acid (LTA), which binds specifically to Toll-like receptor 2 (TLR2) on human macrophages. This phenomenon has the potential to be exploited for the design of biomimetic peptides for antibacterial application. In this study, we have derived peptides from sequences present in human TLR2 that bind to LTA with high affinity. In silico approaches including molecular modelling, molecular docking, molecular dynamics, and thermodynamics have enabled the identification of these crucial binding amino acids, the design of four novel biomimetic TLR2-derived peptides and their LTA binding potential. The outcomes of this study have revealed that one of these novel peptides binds to LTA more strongly and stably than the other three peptides and has the potential to enhance LTA targeting and bacterial cell penetration.

## 1. Introduction

The World Health Organization has declared antimicrobial resistance as among the top 10 human health threats worldwide. Bacterial infection as a result of antimicrobial resistance has caused of millions of deaths all over the world and is predicted to cause millions more, especially in countries where clean water, proper sanitation, and infection prevention and control are lacking [1].

Current treatments of bacterial infections have suboptimal targeting of bacterial cells, which results in elevated exposure to healthy cells and probiotics, increased frequency of administration, lack of association with the bacteria, minimal concentration of treatment at the bacterial infection site and prolonged treatment period [2,3]. Lack of effective antimicrobials puts the success of current treatments at greater risk [1]. There is slow progress in the discovery and development of new antimicrobial agents, exacerbating the burden of antimicrobial resistance on morbidity and mortality rates [4]. Hence, innovative strategies for targeted treatment are necessary to curb the current resistance of bacteria to antimicrobials.

Researchers are harnessing biomimetic strategies for enhanced targeting of therapies for various diseases, including cancer [5], viruses [6], fungal infections [7], central nervous system diseases [8], and bacterial infections [9]. Biomimetic strategies in drug delivery take advantage of mechanisms of interaction between living cells and biological pathways, activities and structures and have been used to enhance the targeting, pharmacokinetics, circulation time, antibacterial activity and biosafety of nanoparticle formulations [9,10]. Biomimetics have been used to enhance the synthesis and drug delivery of nanosystems for antibacterial application in various ways [11]. Some of them include the use of biomasses from protein, fungi, and plants for the synthesis of antibacterial nanoparticles for their beneficial capping and bioreduction activities [12,13], while others involve mimicry of cell surface structures [14] and extracellular matrix structures [15] for enhanced pharmacokinetic parameters, disruption of biofilm and bacterial toxin neutralization [16], and prevention of biofilm formation and bacterial adhesion on surfaces of biomedical device implants [17], among other benefits.

In this study, we have created a novel biomimetic approach that uses nature’s binding specificity to design biomimetic peptides for targeting Gram-positive bacteria, specifically *Staphylococcus aureus* and methicillin-resistant *Staphylococcus aureus* (MRSA). When a host is infected by Gram-positive bacteria, as a host defense mechanism, the innate immune system becomes activated when membrane-bound pattern recognition receptors (PRRs) of human macrophages such as Toll-like receptor 2 (TLR2) recognize bacterial lipoteichoic acid (LTA) on Gram-positive bacterial cell walls [18]. The novelty of our strategy lies in the exploitation of the natural and unique affinity of the convex surface of TLR2 for LTA on Gram-positive bacterial cell walls [19,20]. We have taken advantage of this unique phenomenon and derived four peptides from naturally occurring amino acid sequences present in human TLR2 that bind to and interact with LTA with high affinity, which can be used to design targeted delivery systems. Due to the biomimetic components of these peptides, they may possess beneficial properties as pathogen recognition pattern, cell-penetrating peptides, which include enhanced targeting to LTA, improved antibacterial efficiency, biocompatibility, and biodegradability. This is the first report of such a biomimetic approach, which includes an in silico assessment of the binding potential of these biomimetic TLR2-derived peptides (BTp1, BTp2, BTp3, BTp4) to LTA. Based on the stability and strength of binding of these peptides to LTA, they may have the potential to be used in the surface modification of antibacterial nanoparticles for enhanced targeting of LTA on bacterial cell walls and bacterial cell penetration to deliver antibacterial drugs during bacterial infection.

## 2. Materials and Methods

### 2.1. Receptor and Ligand Acquisition/Design and Preparation

The 3D crystal structure of human TLR2 was acquired from RSCB Protein Data Bank [21] (PDB: 6nig), while the 3D structure of LTA was acquired from PubChem [22] (ID: 137349712). The geometry of the LTA ligand was optimized using Avogadro [23], and UCSF Chimera software [24] was used to add hydrogens and charge to the ligand. The peptides were designed and optimized using Schrödinger Maestro software [25] and prepared for molecular docking using UCSF Chimera software (Version 1.14).

### 2.2. Molecular Docking

Molecular docking is a frequently used approach in structure-based drug design to predict the most favorable binding conformations of ligands to target molecules [26]. Herein, molecular docking was applied in two separate instances: to predict the conformation of LTA that binds with the highest affinity to TLR2; and to predict the preferable conformations and binding affinities of novel peptides to LTA. The software that were used to prepare molecules and perform docking include UCSF Chimera, AutoDock MGL tools and AutoDockVina [27] and Raccoon [28], using default parameters. The ligand conformations that bound with strongest affinity (kcal/mol) were thereafter subjected to molecular dynamic simulations.

### 2.3. Molecular Dynamics Simulation

To provide a more in-depth understanding of the physical movements between, firstly, LTA and TLR2, and secondly, the peptides and LTA, molecular dynamic simulations were performed on the complexes for 10 and 500 ns, respectively. The PMEMD engine of the Amber 18 suite was used to perform all simulations [29]. Atomic partial charges were produced by ANTECHAMBER using General Amber Force Field commands. The Leap module solvated the systems within a water box (10 Å TIP3P). To neutralize the systems, Na^+^ or Cl^−^ counter-ions were introduced. An initial 2500 step minimization was carried out, with a restraint potential of 500 kcal/mol. A 1000 step full minimization was carried out thereafter, without restraints. This was followed by gradual heating to 300 K for 50 ps, such that each system provided a fixed number of atoms and volume. A potential harmonic restraint of 10 kcal/molÅ^−2^ was performed on the solutes of the systems. Thereafter, systems were equilibrated for 500 ps. An isobaric-isothermal ensemble (NPT) was imitated throughout the simulations (1 bar system pressure, constant number of atoms), using the SHAKE algorithm to constrain hydrogen bonds and a Langevin thermostat with collision frequency of 1 ps^–1^. 

### 2.4. Post-Dynamic Analyses

The root-mean-square fluctuation (RMSF), root-mean-square deviation (RMSD) and thermodynamic energy of the systems were calculated to further analyze the systems’ stability and energy inputs. The co-ordinates of each system were recorded every 1 ps and the trajectories were analyzed via the AMBER 18 CPPTRAJ module [30]. 

Thermodynamic Energy Calculation and Per-Residue Energy Decomposition Analysis were conducted to establish the free energy of binding (ΔG_bind_) of the novel peptides to LTA. Molecular mechanics integrated with the generalized Born surface area continuum solvation (MM/GBSA) method [31] were used.

The molecular dynamics simulation generated a trajectory of 500,000 snapshots, which were averaged to produce ΔG_bind_. The binding affinities between the peptides and LTA may be defined as Equations (1)–(5):

(1) $$\Delta G_{\mathrm{bind}}=G_{\mathrm{complex}}-G_{\mathrm{receptor}}-G_{\mathrm{ligand}}$$

(2) $$\Delta G_{\mathrm{bind}}=E_{\mathrm{gas}}+G_{\mathrm{sol}}-\mathrm{TS}$$

(3) $$E_{\mathrm{gas}}=E_{\mathrm{int}}+E_{\mathrm{vDW}}+E_{\mathrm{ele}}$$

(4) $$G_{\mathrm{sol}}=G_{\mathrm{GB}}+G_{\mathrm{SA}}$$

(5) $$G_{\mathrm{SA}}=\gamma \mathrm{SASA}$$

where:

E_ele_ Electrostatic potential energy from Coulomb forces
E_gas_ Gas-phase energy (based on FF14SB force field terms)
E_int_ Internal energy
E_vdW_ van der Waals energy
G_sol_ Solvation free energy
G_GB_ Polar solvation energy
G_SA_ Non-polar solvation energy
S Total entropy of solute
SASA Solvent accessible surface area (water probe radius of 1.4 Å)
T Total entropy of temperature

A per-residue energy decomposition was also calculated to predict the energy contribution of each amino acid residue to the total ΔG_bind_ at the peptide binding site, using the AMBER14 MM/GBSA approach.

## 3. Results and Discussion

### 3.1. Binding Affinity of TLR2 to LTA

**Figure 1:**
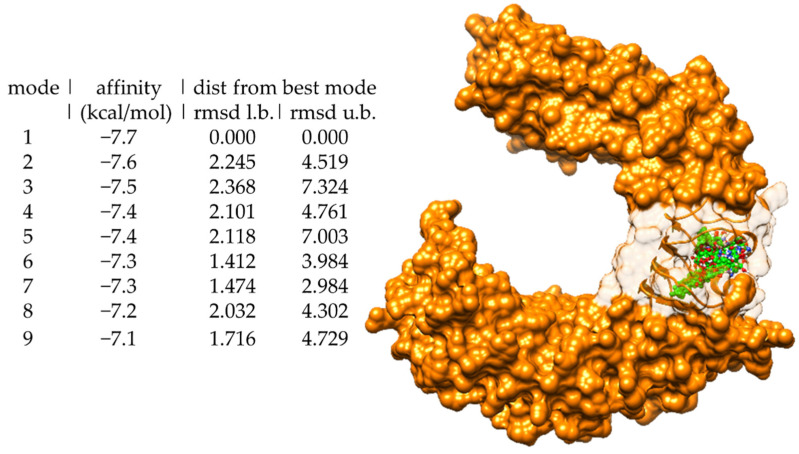
Binding affinities of nine best poses of LTA (**left**) and all nine conformations superimposed in the binding site of TLR2 (**right**).

Molecular docking of LTA to the binding site on human TLR2 revealed the nine best poses of LTA bound to TLR2, with their respective binding affinities (Figure 1). The conformation of LTA that bound with highest affinity to TLR2 and with zero RMSD is indicated by mode number 1 in Figure 1, with binding energy of −7.7 kcal/mol. 

### 3.2. Molecular Dynamics of TLR2/LTA Complex for Acquisition of Binding Site Amino Acid Residues

**Figure 2:**
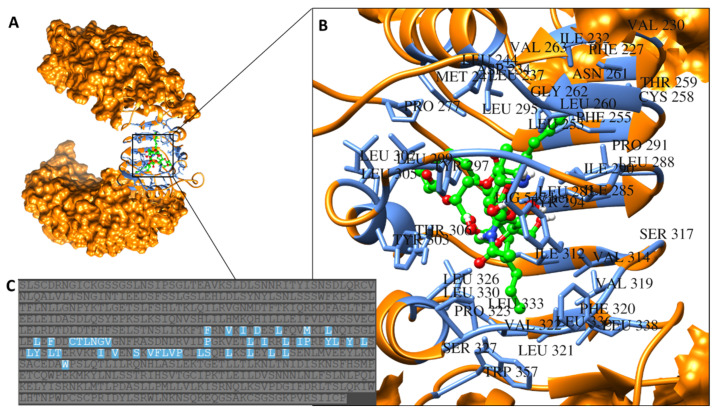
(**A**) Location of LTA (green) binding on TLR2. (**B**) Specific binding site amino acid residues on TLR2 (blue) bound by LTA (green). (**C**) Sequence of TLR2 with specific binding site residues highlighted in blue correlating with residues identified in (**B**).

The docking pose of LTA with the strongest binding affinity (−7.7 kcal/mol) to TLR2 was selected to be simulated with TLR2 for 10 ns to establish the most accurate prediction of the interaction between LTA and TLR2. After simulating the complex, binding site amino acid residues were identified using Schrödinger Maestro and UCSF Chimera software (Figure 2).

### 3.3. Design of Potential Biomimetic TLR2-Derived Targeting Peptides

Using the amino acid residues of TLR2 that bind with LTA (highlighted in Figure 2), we identified four regions of significance for binding and used their amino acid sequences to design peptides with sequences as follows: CTLNGV, RRLHIPRF, YDLLYSLT and SKVFLVP. Upon drawing 3D structures of the novel biomimetic TLR2-derived peptides (BTp1, BTp2, BTp3, and BTp4, respectively) using Schrödinger Maestro software, their geometries were optimized, and structures minimized.

### 3.4. Binding Affinity of Biomimetic TLR2-Derived Peptides to LTA

Thereafter, molecular docking of each peptide to LTA revealed the nine best poses of LTA bound to each peptide, with their respective binding affinities (Table 1). The best binding pose of BTp1 bound with −2.5 kcal/mol to LTA, BTp2 with −3.4 kcal/mol, BTp3 with −2.9 kcal/mol and BTp4 with −3.8 kcal/mol. Therefore, BTp2 and BTp4 bind with strongest affinity to LTA, compared to BTp1 and BTp3.

### 3.5. Molecular Dynamics, Stability, Thermodynamics and Per-Residue Binding Free Energy of BTp /LTA Complexes

**Figure 3:**
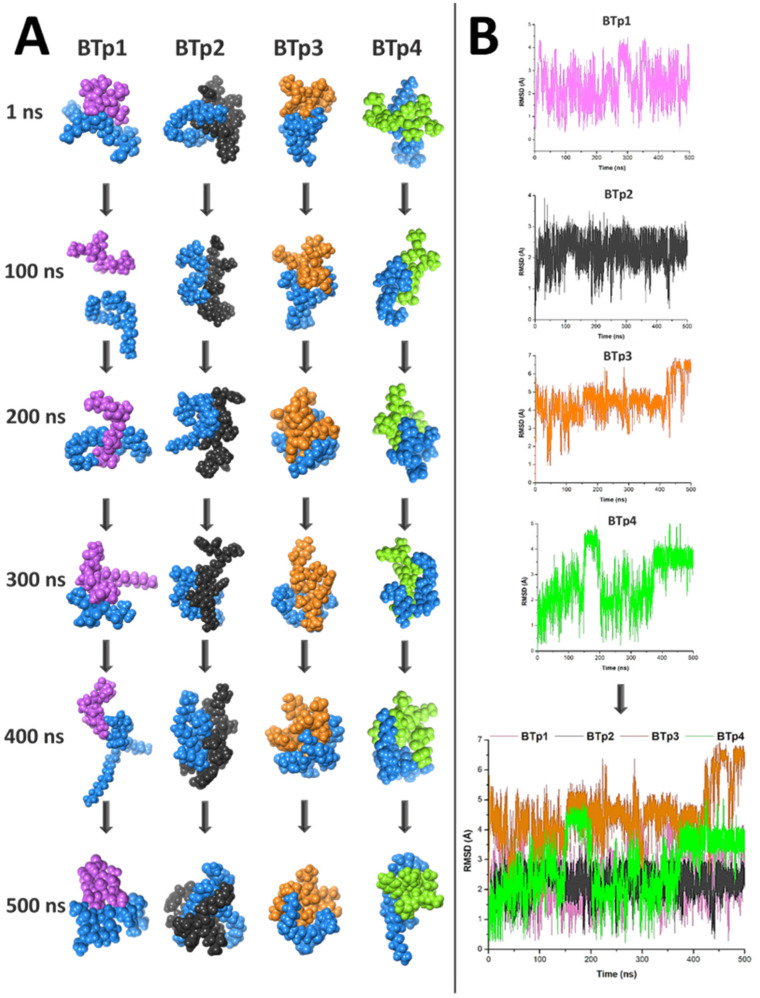
Trajectory analyses of four novel peptides bound to lipoteichoic acid. (**A**) Snapshots during 500 ns simulations of each peptide bound to lipoteichoic acid at 100 ns intervals. (**B**) Graphs depicting RMSD calculations of each of the four simulations and superimposed in the last graph.

The conformations of each peptide that bound with strongest affinity to LTA (highlighted in bold and cyan in Table 1) from molecular docking studies were then subjected to molecular dynamics simulations for 500 ns each, to validate the docking scores and to provide a more in depth understanding of the atomic interactions between LTA and the peptides. Figure 3A illustrates snapshots at 100 ns intervals of each of the four novel peptides simulated with LTA for 500 ns each. The graphs in Figure 3B depict RMSD calculations of simulations of each novel peptide bound to LTA, and finally superimposed onto one graph for comparison purposes. In addition, MM/GBSA thermodynamic energy calculations and per-residue energy decomposition of every complex are provided in detail.

From Figure 3, information regarding the stability of the four simulations can be deduced. Figure 3A confirms that BTp1 and LTA bound unstably, as evident in the 100 ns snapshot when they detached from each other and in the 400 ns snapshot (Figure S1) when they were only bound via a hydrogen bond between THR2 and O12, and a carbon hydrogen bond between CYS1 and O12, of BTp1 and LTA, respectively. RMSD calculations (Figure 3B) confirmed instability of the complex as the system did not reach convergence throughout the simulation and the energy fluctuated beyond a 2 Å range (~4 Å). The energy fluctuation of each residue throughout the simulation was established via RMSF calculations, which showed that during the 500 ns simulation of the BTp1 and LTA bound complex, GLY5 of BTp1 was the most stably bound residue as its energy fluctuated the least, while the energy of VAL6 fluctuated the most (Figure S5). 

**Figure 4:**
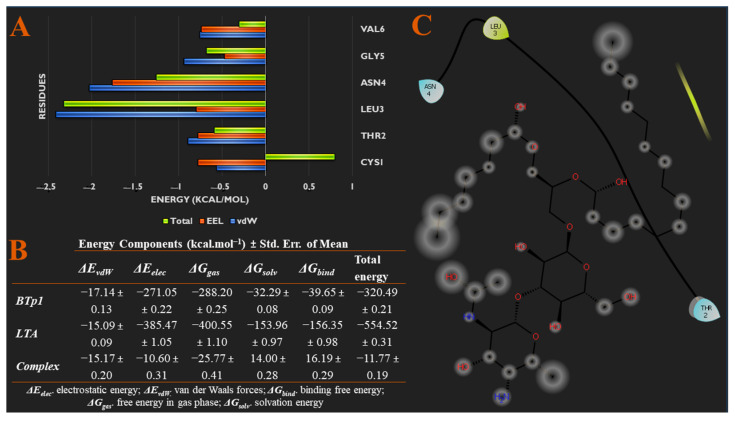
(**A**) Per-residue energy decomposition analysis showing specific energy contributions of each amino acid residue of BTp1 that contributes to binding. (**B**) Different energy contributions of each component of the system via MM/GBSA thermodynamics. (**C**) Binding interaction diagram of BTp1 to residues of TLR2.

Figure 4A,C present specific energy contributions of each amino acid residue of BTp1 that contributes to binding. Van der Waals (vdW) contribution to binding is predominantly from LEU3 and ASN4, while electrostatic (EEL) energy contribution stems from ASN4. These two residues are significant to the binding of BTp1 to LTA. Finally, Figure 4B also provides the different energy contributions of each component of the system, which shows the total binding free energy of the bound complex is −11.77 kcal/mol.

**Figure 5:**
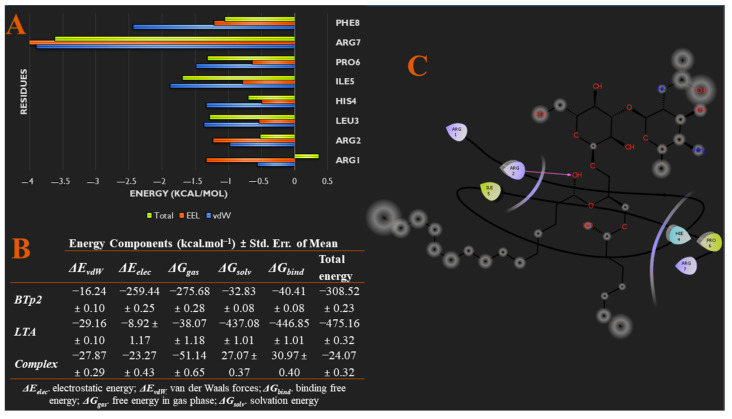
(**A**) Per-residue energy decomposition analysis showing specific energy contributions of each amino acid residue of BTp2 that contributes to binding. (**B**) Different energy contributions of each component of the system via MM/GBSA thermodynamics. (**C**) Binding interaction diagram of BTp2 to residues of TLR2.

On the other hand, Figure 3A also shows that BTp2 and LTA bound tightly and consistently throughout the simulation, compared to BTp1. The second RMSD graph in Figure 3B indicates that after 454 ns, the system reached convergence and energy fluctuations remained within a 2 Å range. Particularly, at 400 ns (Figure S2), just before the system reached convergence, multiple interactions between BTp2 and LTA were observed. Hydrogens bonds were formed between ARG1 and O12, ARG2 with O12, H4 and H54, HIS4 with O10, ILE5 with H5, H42 and H43, ARG7 with O8, and PHE8 with O8, of BTp2 and LTA, respectively. In addition, HIS4 of BTp2 interacts with H67 of LTA via a Pi-Sigma bond. These data support the results from molecular docking studies, which demonstrated stronger binding of BTp2 to LTA, than BTp1. From Figure S5, we can see that the ARG7 of BTp2 fluctuated the most in the system, along with ARG1 and ARG2, with the highest RMSF values, while PRO6 fluctuated the least. Further analyses show in Figure 5A,C specific energy contributions of each amino acid residue of BTp2 that contributes to binding. From this, most of the vdW and EEL energy contribution to binding is predominantly from ARG7, which is in accordance with results of RMSF calculations. Furthermore, Figure 5B also provides the different energy contributions of each component of the system, which shows the total binding free energy of the bound complex is −24.07 kcal/mol. This is stronger than binding of BTp1 to LTA, which supports binding affinity results from molecular docking studies that showed BTp2 binds better to LTA than BTp1.

The trajectory of the BTp3/LTA system in Figure 3A showed that BTp3 remained bound to LTA throughout the 500 ns simulation, more strongly than BTp1 but less than BTp2. However, the third RMSD analysis in Figure 3B showed that the system did not reach convergence, as evident by the large spike in energy after 421 ns. Moreover, energy deviations were greater than 2 Å (~6 Å), indicating the instability of the complex. In addition, Figure S3 clearly shows that at 400 ns, the complex is stable with several bonds and interactions, while at 500 ns, many of the interactions are lost. In more detail, the 400 ns snapshot shows a carbon hydrogen bond between TYR1 and H54, and alkyl interactions between LEU3 with C23 and C35, and LEU7 with C23 and C31, of BTp3 and LTA, respectively, while the 500 ns snapshot only shows carbon hydrogen bond interactions between SER6 and H56, and SER6 and H57, of BTp3 and LTA, respectively.

**Figure 6:**
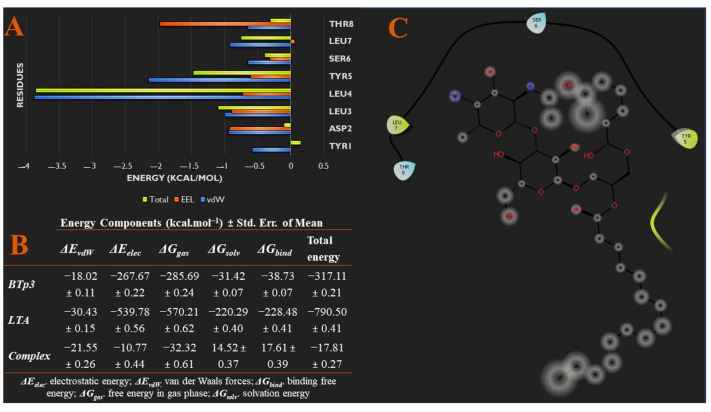
(**A**) Per-residue energy decomposition analysis showing specific energy contributions of each amino acid residue of BTp3 that contributes to binding. (**B**) Different energy contributions of each component of the system via MM/GBSA thermodynamics. (**C**) Binding interaction diagram of BTp3 to residues of TLR2.

Furthermore, Figure 6A,C show specific energy contributions of each amino acid residue of BTp3 that contributes to binding. Most of the EEL energy contribution to binding is from THR8, while vdW contribution is mainly from LEU4. Furthermore, Figure 6B also shows the total binding free energy of the bound complex is −17.81 kcal/mol, which is stronger than binding of BTp1 to LTA, but less than that of BTp2.

**Figure 7:**
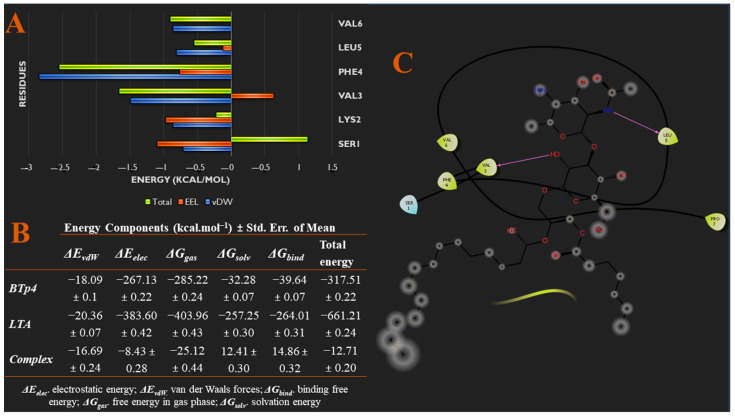
(**A**) Per-residue energy decomposition analysis showing specific energy contributions of each amino acid residue of BTp4 that contributes to binding. (**B**) Different energy contributions of each component of the system via MM/GBSA thermodynamics. (**C**) Binding interaction diagram of BTp4 to residues of TLR2.

From molecular docking studies, BTp4 bound the strongest to LTA, compared to the other peptides. This was supported by the snapshots of the 500 ns trajectory of BTp4 bound to LTA in Figure 3A, which showed that the complex remained bound throughout the entire simulation. In addition, the fourth RMSD graph in Figure 3B indicates that the system reached convergence after 480 ns as fluctuations remained within a 2 Å range. After reaching convergence, at 500 ns (Figure S4), an alkyl interaction exists between VAL3 and C31, and hydrogen bonds are formed between VAL3 with H5 and H10, PHE4 and H55, LEU5 with H4, H54 and H57, and 2 hydrogen bonds between PRO7 and O12, of BTp4 and LTA, respectively. In addition, Figure 7A,C show specific energy contributions of each amino acid residue of BTp4 that contributes to binding. Most of the EEL energy contribution to binding is from SER1 and LYS2, while vdW and total energy contribution is mainly from PHE4. Furthermore, Figure 7B also shows the total binding free energy of the bound complex is −12.71 kcal/mol, which is still less than binding of BTp2 to LTA.

Taken together, from the initial molecular docking studies, BTp2 and BTp4 showed the highest potential to bind to LTA, with stronger binding affinities than BTp1 and BTp3. After molecular dynamics simulations and post-analyses, only complexes of BTp2 and BTp4 bound to LTA reached convergence, which supported data from molecular docking studies. From this, the most stable peptide that bound to LTA during the 500 ns simulation was BTp2, in terms of RMSD calculations (Figure 3B), as the energy fluctuations were within a ~2 Å range throughout the 500 ns simulation. All other peptides bound to LTA with large energy fluctuations and overall higher energy averages during the simulations. Furthermore, BTp2 binds to LTA with the most negative total binding free energy of −24.07 kcal/mol, compared to all other peptides (BTp2 > BTp3 > BTp4 > BTp1), which suggests that this is the most favorable peptide/LTA bound state. A similar study in rohu fish was conducted to understand the binding between LTA and rohu TLR2 where the binding was found to be −1.92 kcal/mol with only one hydrogen bond present in the interaction plot [32]. Fish TLRs are structurally homologus to human TLRs with a structural sequence identity of ~30–70% [33]. In regard to this, our four novel proposed biomimetic TLR2-derived peptides have stronger binding affinity and more hydrogen bonds interacting between peptides residues and LTA suggesting their competitive binding to LTA.

### 3.6. Lipophilicity of BTp2

Lipophilicity influences the absorption, distribution, metabolism and excretion (ADME) properties of a compound. The log*p* value gives an idea as to whether or not a compound will penetrate living tissue—a positive log*p* value suggests that the compound is more lipophilic and will be able to penetrate cell membranes [34,35]. Using the online tool called SwissADME [36], BTp2 was found to have a positive log*p* value of 1.44 and can therefore pass through cell membranes [35]. Hence, BTp2 not only has the potential to target LTA, but also the potential to be a cell-penetrating peptide. This property will enhance the cell penetration capability of the peptide if used for nanoparticle surface modifaction or coating a drug delivery system. 

## 4. Conclusions

Biomimicry has been applied in drug delivery to benefit from the advantages that come with natural biological pathways [10]. In this study, we aimed to design four biomimetic peptides derived from human TLR2 to evaluate their mimicry resmblelance to human TLR2 and target bacterial LTA as well as their possible cell-penetrating peptide properties. These targeting peptides can be used to surface modify nanodrug delivery systems for enhanced targeting of LTA on bacterial cell walls and improved delivery of antibacterial treatments. This study was a preliminary phase of this evaluation, to determine the potential of these novel peptides to bind to LTA and remain stably bound. However, previous literature reporting compounds identified from in silico approaches has demonstrated successful progression in which the experimental findings concured with the data from molecular modeling [37,38,39,40]. Molecular docking studies, molecular dynamic simulations, post-analyses and thermodynamic energy calculations have shown that all four peptides bind to LTA, but BTp2 and BTp4 bind more strongly than the others. From our results, we have established that BTp2 (RRLHIPRF) bound to LTA more strongly and stably than the other three peptides, and possesses favourable lipophilic properties to enable its cell-penetrating functions. This peptide, therefore, has the potential to target and bind to LTA on bacterial cell walls and permeate the cell membrane, and can be used to surface modify nanodrug delivery systems for enhanced bacterial cell targeting, penetration and improved delivery of antibacterial agents. This serves as a platform for further formulation work into enhanced, targeted nano-antibiotic delivery systems for the treatment of Gram-positive bacterial infection.

## Figures and Tables

**Table 1:** Binding affinity of the top nine poses of four novel peptides to lipoteichoic acid.

Peptide Bound to Lipoteichoic Acid	Binding Affinity			
BTp1: CTLNGV 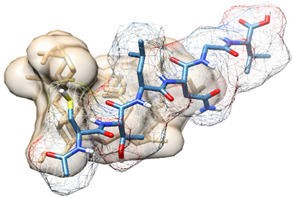	Mode	Affinity (kcal/mol)	Dist from rmsd l.b.	Best mode rmsd u.b.
	**1**	**−2.5**	**0**	**0**
	2	−2.4	9.222	12.669
	3	−2.3	5.016	7.706
	4	−2.2	8.403	13.391
	5	−2.2	8.094	12.23
	6	−2.2	9.072	14.517
	7	−2.2	9.339	13.51
	8	−2.2	8.196	11.353
	9	−2.1	2.631	8.354
BTp2: RRLHIPRF 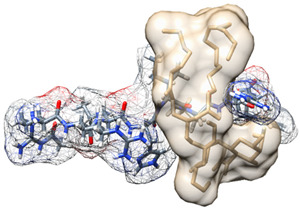	Mode	Affinity (Kcal/mol)	Dist from rmsd l.b.	Best mode rmsd u.b.
	**1**	**−3.4**	**0**	**0**
	2	−3.4	3.659	10.855
	3	−3.4	2.62	5.43
	4	−3.3	1.369	2.413
	5	−3.3	5.024	9.196
	6	−3.2	3.682	11.033
	7	−3.2	4.461	10.666
	8	−3.2	1.816	3.079
	9	−3.2	5.128	8.731
BTp3: YDLLYSLT 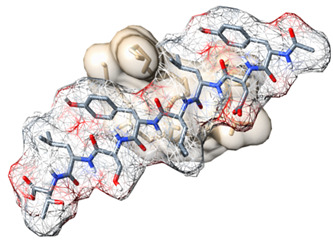	Mode	Affinity (Kcal/mol)	Dist from rmsd l.b.	Best mode rmsd u.b.
	**1**	**−2.9**	**0**	**0**
	2	−2.8	2.658	5.748
	3	−2.8	1.639	4.576
	4	−2.7	4.626	9.394
	5	−2.7	8.39	12.838
	6	−2.7	5.11	9.703
	7	−2.7	8.117	12.937
	8	−2.6	3.682	8.654
	9	−2.6	4.175	8.33
BTp4: SKVFLVP 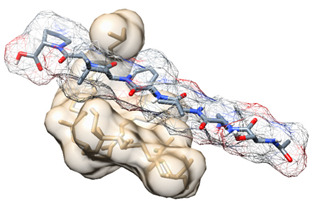	Mode	Affinity (Kcal/mol)	Dist from rmsd l.b.	Best mode rmsd u.b.
	**1**	**−3.8**	**0**	**0**
	2	−3.7	3.574	6.868
	3	−3.7	1.862	2.555
	4	−3.6	2.005	3.056
	5	−3.6	2.577	3.93
	6	−3.6	3.501	7.01
	7	−3.5	2.83	6.586
	8	−3.5	7.209	12.029
	9	−3.4	1.885	3.139

## Data Availability

All data produced from this study may be requested from Prof Thirumala Govender (govenderth@ukzn.ac.za). UCSF Chimera software may be downloaded from https://www.cgl.ucsf.edu/chimera/download.html (Accessed Date 16 August 2021), Avogadro software from https://avogadro.cc/ (Accessed Date 16 August 2021), Schrödinger Maestro software from https://www.schrodinger.com/products/maestro (Accessed Date 16 August 2021), AutoDock MGL Tools from http://mgltools.scripps.edu/downloads (Accessed Date 16 August 2021),, AutoDock Vina from http://vina.scripps.edu/download.html (Accessed Date 16 August 2021), and Raccoon from http://autodock.scripps.edu/resources/raccoon (Accessed Date 16 August 2021).

## Supplementary Materials

The following are available online at https://www.mdpi.com/article/10.3390/biomedicines9081063/s1, Figures S1–S4: Interaction bonds between all biomimetic TLR2-derived peptides with LTA; Figure S5: Mean energy fluctuations (RMSF calculations) of each peptide over 500 ns.
