# Supplementary material for: Novel Biomimetic Human TLR2-Derived Peptides for Potential Targeting of Lipoteichoic Acid: An In Silico Assessment
Source: Biomedicines. 2021 Aug 21;9(8):1063. doi: 10.3390/biomedicines9081063 (PMC8391229; doi:10.3390/biomedicines9081063)
Supplement: Supplementary file 1 [file biomedicines-09-01063-s001.zip › biomedicines-1350353-supplementary.pdf]

Electronic Supplementary Information

Novel biomimetic human TLR2-derived antimicrobial peptides for potential targeting of lipoteichoic acid: An *in silico* assessment.

Nikita Devnarain, Ayman Waddad, Beatriz G. de la Torr, Fernando Albericio and Thirumala Govender

Interaction bonds between all biomimetic TLR2-derived peptides with LTA and Mean energy fluctuations (RMSF calculations) of each peptide over 500 ns

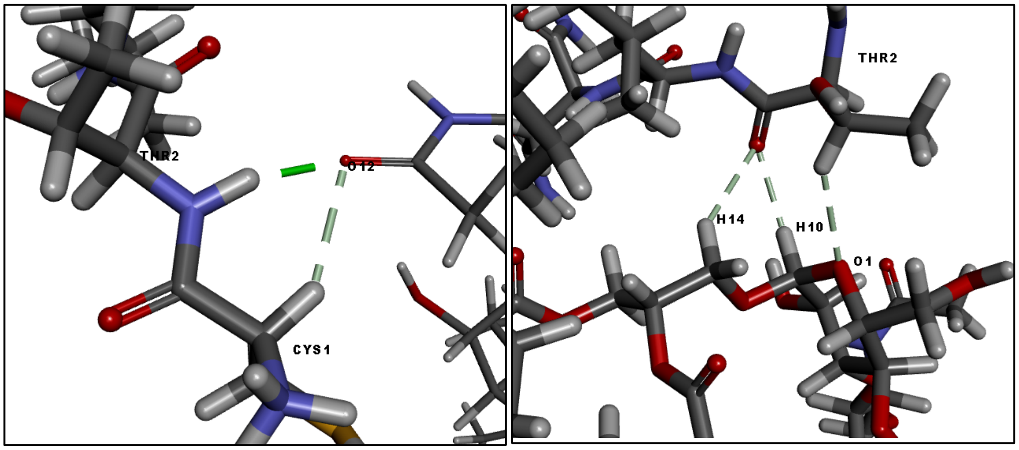

Figure S1. BTp 1 bound to LTA at 400 ns (left) and 500 ns (right).

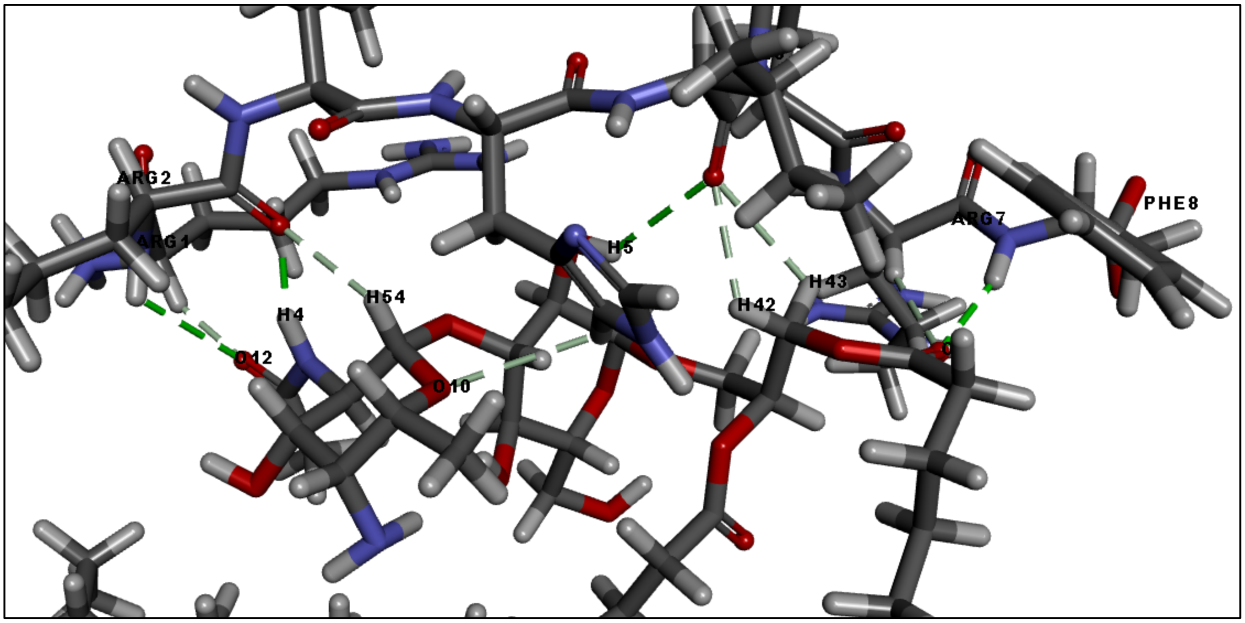

Figure S2. BTp 2 bound to LTA at 400 ns.

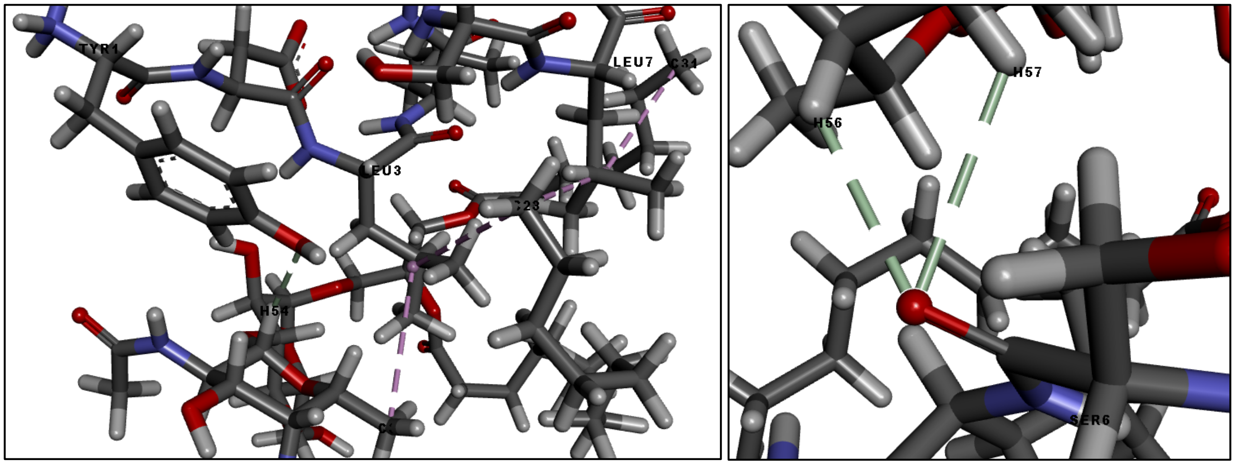

Figure S3. BTp 3 bound to LTA at 400ns (left) and 500 ns (right)

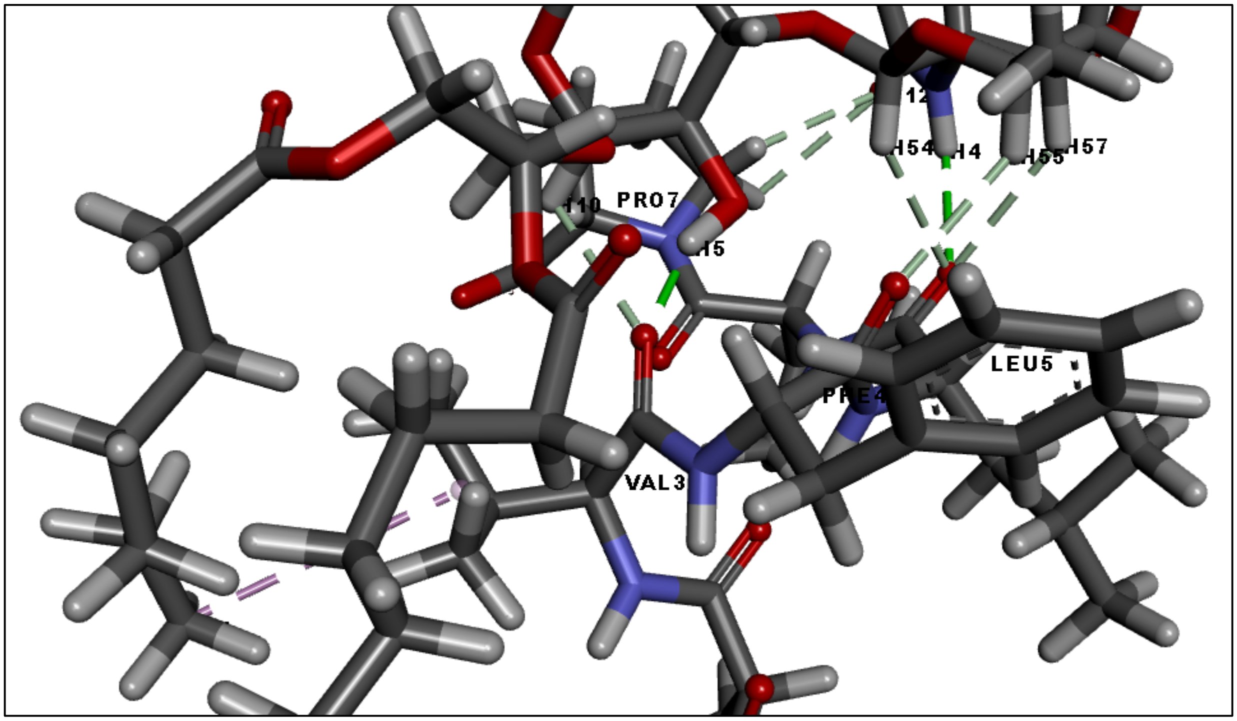

Figure S4. BTp 4 bound to LTA at 500 ns

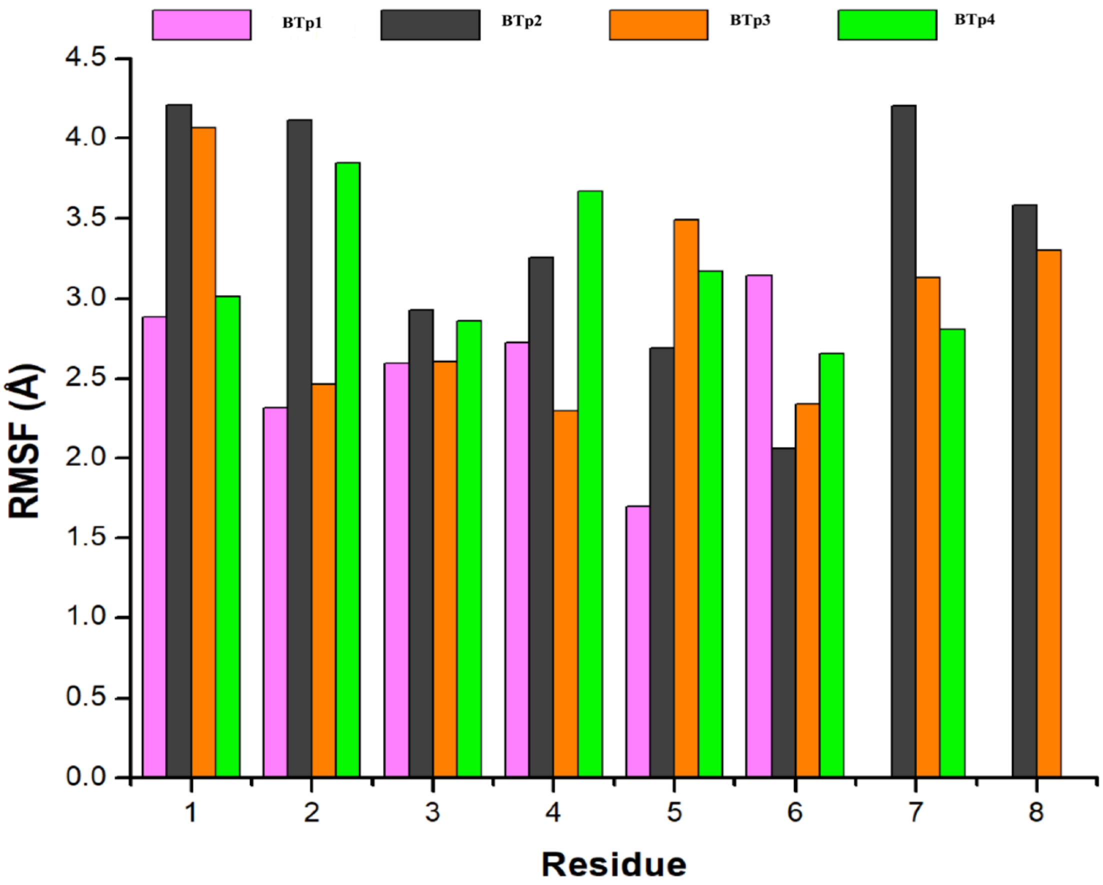

Figure S5. Mean energy fluctuations (RMSF calculations) of each peptide over 500 ns.
